# Intersections of climate and society: cold stress vulnerability in Northern Norway

**DOI:** 10.1007/s00484-026-03335-w

**Published:** 2026-10-08

**Authors:** Elena Grigorieva, John Walsh, Vladimir Alexeev

**Affiliations:** 1 Department of Geography, Humboldt Universität zu Berlin (HU), Berlin, 10099 Germany; 2 International Arctic Research Centre, University of Alaska Fairbanks, Fairbanks, AK USA

**Keywords:** Extreme cold, Universal Thermal Climate Index, Social vulnerability index, Adaptive capacity, Northern Norway

## Abstract

Extreme cold profoundly shapes health, livelihoods, and social well-being in northern communities, yet research on social vulnerability to such conditions remains limited. This study provides a framework for integrating social, demographic, infrastructural, and institutional dimensions into an assessment of vulnerability to extreme cold in northern Norway. It utilizes the Extremely Cold Social Vulnerability Index (ECSVI, ranging from 0 to 1) derived from the Universal Thermal Climate Index (UTCI), and indices of social sensitivity and social adaptivity for 83 communities across Nordland, Troms, and Finnmark in Northern Norway during 2000–2019. Finnmark experienced substantially greater cold exposure than Nordland and Troms, although annual exposure to UTCI < − 27 °C declined from 1011 to 883 h between 2000 and 2009 and 2010–2019. ECSVI showed marked regional differences, with Finnmark remaining the most vulnerable county (0.69 and 0.67 in two decades), compared with substantially lower values in Troms and Nordland. At the community level, Social Sensitivity ranged from 0.27 to 0.61 and Social Adaptivity from 0.39 to 0.64. Low-income households (*r* = 0.64), foreign-born population (*r* = 0.62), and income inequality (*r* = 0.60) showed the strongest positive associations with Social Sensitivity. Infrastructure metrics such as connectivity to the municipal water supply (*r* = 0.61) and roads with street lighting (*r* = 0.46) were the highest correlators with Social Adaptivity. Overall, the results demonstrate that vulnerability to extreme cold reflects the combined effects of climatic exposure and socioeconomic conditions, while stronger adaptive capacity can reduce vulnerability. By quantifying vulnerability across and within regions, this study offers actionable insights for targeted adaptation and resilience planning.

## Introduction

**Fig. 1:**
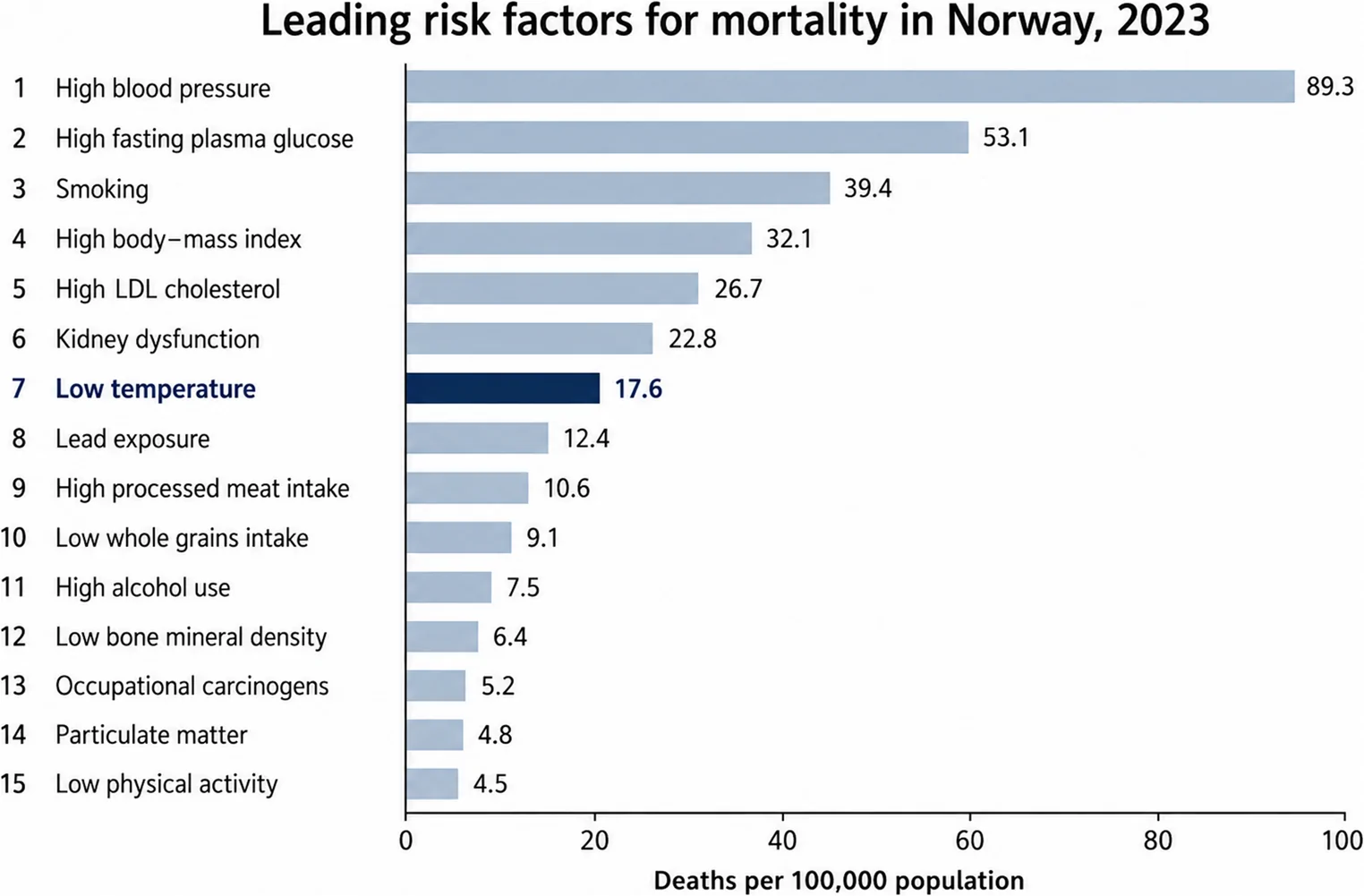
Leading risk factors for mortality in Norway in 2023 (%). *Source*: Institute for Health Metrics and Evaluation (IHME), 2024

The Arctic is experiencing some of the earth’s fastest environmental changes, with warming rates three to four times higher than the planetary average (Rantanen et al. 2022). This rapid warming drives more frequent and intense climate hazards, including permafrost degradation, flooding, and extreme weather, threatening ecosystems, infrastructure, and human communities (Walsh 2021; Overland 2024). Such transformations carry profound social, economic, cultural, and security implications, compounded by the Arctic’s interconnectedness with global systems (Malik et al. 2025; Naik et al. 2025). Cold vulnerability emerges from interactions between harsh climates, settlement patterns, cultural practices, and social systems (Risvoll et al. 2022). The Institute for Health Metrics and Evaluation (2024) places low temperature as the 13th leading risk factor of death globally (versus 25th for high temperature), and the 7th leading risk factor in Norway, where heat ranks among the least significant of over 50 factors (Fig. 1). Since the six leading risk factors in northern Norway are health-related, low temperature is actually the highest-ranking environmental risk factor in the region.

Even in a warming climate, extreme cold events are expected to continue to occur and to pose risks (Hanna et al. 2024). Despite these risks, adaptation to extreme cold remains limited, highlighting the need for targeted research on social vulnerability, resilience, and adaptation to support sustainable development and social equity in Arctic regions (Grigorieva 2024; Tingstad et al. 2025). In particular, effective strategies for adaptation to cold must address persistent inequalities in health, income, and service access while mitigating climate impacts on food, water, energy, and livelihoods.

Ahmed et al. (2024) identified “vulnerability” as the most frequently cited concept in Arctic sustainability studies, emphasizing the link between environmental hazards and social determinants of well-being. Vulnerability is dynamic, multidimensional, and spatially variable, reflecting both external hazards and internal capacities to anticipate, cope with, and recover from impacts (De Pascale 2023; Bronfman et al. 2024). Northern Norway, which contains communities of diverse sizes, accessibility and cultural backgrounds, provides a testbed for assessing the links between environmental hazards and well-being. In Northern Norway, understanding vulnerability requires considering remoteness, population aging, uneven healthcare and infrastructure access, all of which influence capacity to withstand extreme cold.

### Measuring social vulnerability to cold

Social vulnerability arises from community-level social, economic, and institutional characteristics affecting preparation, response, and adaptation to hazards (Cutter et al. 2003). This perspective emphasizes that vulnerability is not solely environmental but is shaped by structural inequalities. The hazards-of-place model (Cutter et al. 2003) links physical risks with socioeconomic conditions, forming the basis for social vulnerability indices. Relevant factors in vulnerability to cold include age, income, education, health, energy access, and infrastructure. While heat-related vulnerability has been widely studied (Bao et al. 2015; Luo et al. 2025), cold-related vulnerability is underexplored, despite cold accounting for higher global mortality than heat (Haman et al. 2022; Fan et al. 2023; Ni et al. 2024). Assessment of cold-related risks requires the integration of extreme-cold metrics into broader disaster and vulnerability frameworks (Lauta et al. 2018).

### Environmental and societal challenges in Northern Norway

Extreme cold significantly affects health, livelihoods, and adaptation in Northern Scandinavia, yet quantitative research on linkages remains limited. Factors such as age, gender, indoor temperature, clothing, and socioeconomic status mediate winter mortality (Rocklöv et al. 2014). Swedish studies show elevated mortality during cold extremes, particularly among older adults (Ni et al. 2024). In Norway, northern municipalities face heightened vulnerability due to economic marginalization and demographic decline, which has been accelerated by youth migration, while urban centers benefit from better service access despite built-environment risks (Holand et al. 2011; Holand and Lujala 2013). Integrating social vulnerability into regional and municipal planning is essential, requiring locally tailored assessments, inter-municipal coordination, and inclusion of socioeconomic indicators (O’Brien et al. 2004; Rød et al. 2025).

Localized, equity-based strategies are critical in Arctic and sub-Arctic regions. Indigenous populations often face inadequate housing and limited healthcare access, heightening vulnerability (Lundblad et al. 2023). Northern Norway faces compounding environmental and social challenges: economic dependence on traditional industries limits diversification and resilience (OECD 2017); remoteness restricts access to healthcare, education, and emergency response, exacerbating risks for elderly and Indigenous populations; and climate change – permafrost thaw, sea-level rise, and extreme weather – threatens infrastructure, livelihoods, and ecosystems (Hanssen-Bauer et al. 2017; Rød et al. 2025).

### Conceptualizing vulnerability

Vulnerability is central to Arctic sustainability research, providing insight into how communities experience, respond and adapt under environmental and social pressures. Adger (2006) defines vulnerability as susceptibility to harm from environmental and social stresses and from a lack of adaptive capacity, while the IPCC (2022) frames it as the predisposition to be adversely affected by climate change, shaped by exposure, sensitivity, and adaptive capacity.

### Adaptation strategies across scales

Adaptation to cold hazards operates at multiple levels. At the community scale, measures include housing insulation, heating maintenance, transport reinforcement, emergency preparedness, and social cohesion (Hovelsrud and Smit 2010; Holand et al. 2011; Lundblad et al. 2023). Indigenous knowledge guides mobility, subsistence, and resource management under extreme conditions (Keskitalo et al. 2016; Rød et al. 2025). Municipal adaptation integrates climate risk into land-use, infrastructure, and healthcare planning (O’Brien et al. 2004; Hovelsrud et al. 2018), while regional and national governance coordinates resources, early warning systems, and long-term policy, aligning with Arctic Council and IPCC frameworks (Keskitalo et al. 2016; IPCC 2022).

In summary, understanding social vulnerability to extreme cold in Northern Scandinavia requires integrating environmental, social, and infrastructural perspectives. The aim of this study therefore is to advance quantitative understanding of social vulnerability to extreme cold in Northern Norway by adapting and applying the Extremely Cold Social Vulnerability Index (ECSVI) to the region. While the ECSVI integrates key physical and social variables such as income, housing quality, health status, and infrastructure (Grigorieva et al. 2024), it has not yet been implemented in Scandinavia, where distinct climatic, social, and infrastructural conditions may shape vulnerability differently from other regions, especially those in middle latitudes. Focusing on the three northernmost Norwegian Arctic counties – Nordland (Bodø), Troms (Tromsø), and Finnmark (Alta) – this study examines how demographic, socioeconomic, and environmental factors influence community sensitivity to extreme cold. By estimating spatial and temporal variations in vulnerability, the research seeks to pinpoint high-risk populations and municipalities, providing actionable insights for targeted adaptation, resilience planning, and locally informed strategies that reduce social vulnerability and enhance the long-term sustainability of Arctic communities.

## Materials and methods

### Study area

**Fig. 2:**
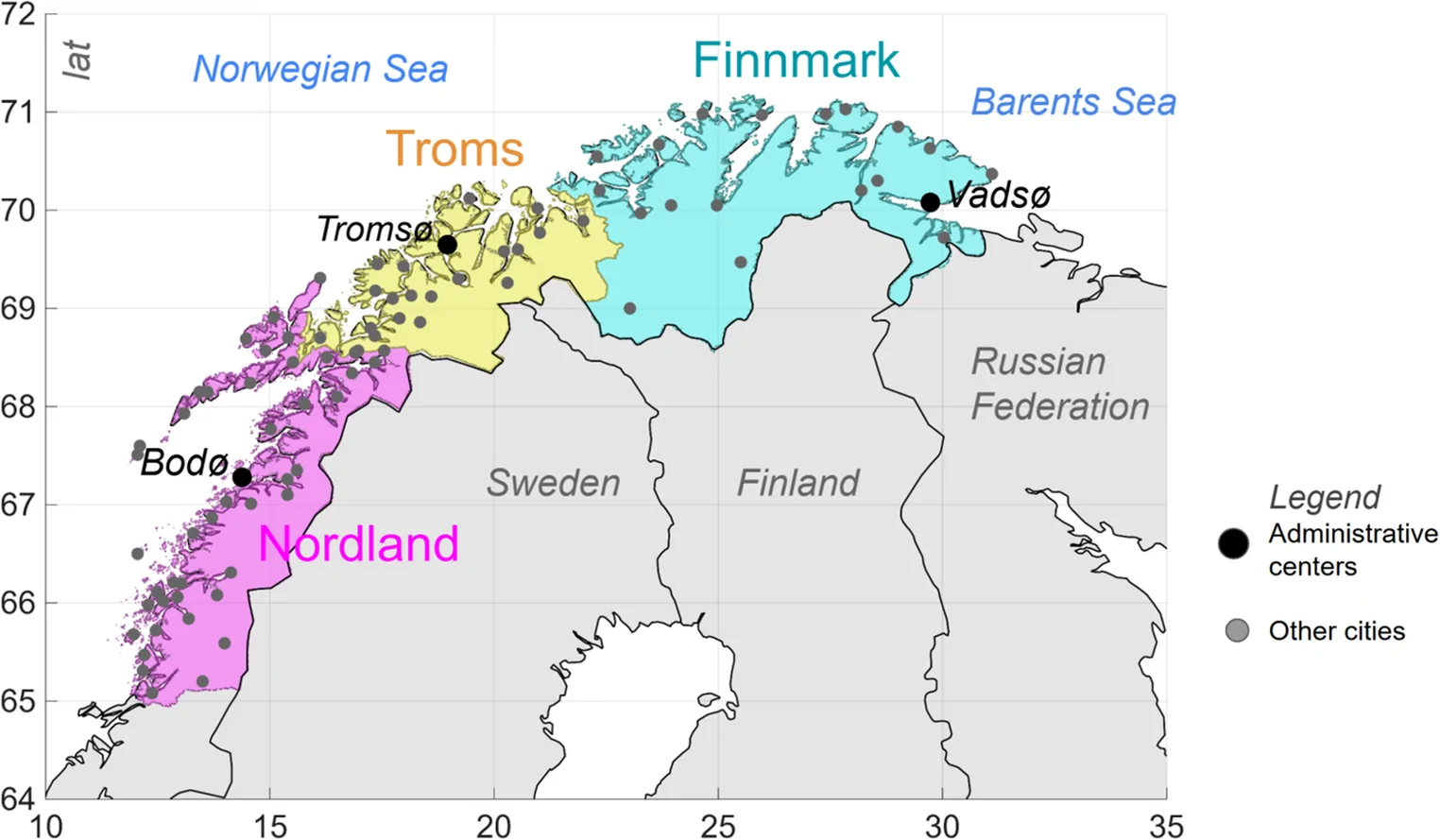
Study area’s three counties in Northern Norway: Bodø (purple), Tromsø (yellow) and Finmark (blue). Dots denote locations of communities for which UTCI and social indicators were evaluated

The study area encompasses Northern Norway, including the three northernmost counties: Nordland, Troms, and Finnmark (Fig. 2), collectively referred to as the Northern, or the Arctic region of Norway. This area extends above the Arctic Circle and covers approximately 35% of the country’s landmass while hosting only about 9.4% of the population (OECD 2017). The region is characterized by a highly varied topography of mountains, fjords, plateaus, and an extensive, fragmented coastline. Settlement patterns reflect these physical constraints, with most communities located along the coast and fewer in the cold, sparsely populated inland plateaus.

Climatically, Northern Norway exhibits pronounced spatial gradients influenced by latitude, altitude, and proximity to the ocean. According to Czekirda et al. (2023), coastal and fjord zones fall within a sub-polar oceanic (Cfc) climate regime, with relatively mild winters and cool summers moderated by maritime influences. Inland and upland regions display subarctic (Dfc) conditions, characterized by long, cold winters and short, cool summers, while high-altitude plateaus approach tundra (ET) or alpine climates, where the warmest month remains below 10 °C. Winter temperatures may drop below − 40 °C in interior valleys, imposing significant constraints on infrastructure and human activities. Observational records indicate increased heavy snowfall and snow accumulation in Troms over recent decades, with projected declines at lower elevations toward the end of the 21st century (Dyrrdal et al. 2020). In Finnmark, Holocene peatland records indicate long-term persistence of permafrost and cold-climate processes that are now being disrupted by ongoing regional warming (Kjellman et al. 2018). These climatic gradients result in heterogeneous exposure to cold stress, necessitating regionally differentiated assessments of social vulnerability.

The total population of Northern Norway is approximately 490,000 inhabitants, with densities as low as 1.6 inhabitants km⁻² in some remote municipalities of Finnmark and North Troms (Angell et al. 2016). Demographic trends indicate persistent population decline and ageing, driven by youth out-migration and low birth rates (Vennerød and Schjøtt-Pedersen 2023). Urban centers such as Tromsø attract younger cohorts, including members of Sámi communities, seeking education and employment (Melhus et al. 2020). Economically, the region depends on natural resource sectors such as fisheries, petroleum, and tourism (OECD 2007, 2017). Access to healthcare, education, and transport infrastructure remains uneven, particularly in remote areas (Angell et al. 2016). Collectively, these geographic, climatic, and demographic conditions shape the spatial patterns of social vulnerability and adaptive capacity that this study seeks to quantify.

### Data

The exposure data were derived from the number of hours with a Universal Thermal Climate Index (UTCI) below ‒27 °C at 83 locales around three counties in Northern Norway (Fig. 2) between 2000 and 2019 and averaged over two decades, 2000‒2009, and 2010‒2019. The UTCI values were obtained from the ERA5-HEAT dataset ‒ a high-resolution (0.25° × 0.25°) gridded reconstruction of surface-level human thermal comfort, provided by the Copernicus Climate Change Service (C3S) (C3S 2020; Di Napoli et al. 2020; Hersbach et al. 2020). For each of the 83 locations, the ERA5-HEAT grid cell with the geographic center nearest to the respective weather station was used in the analysis. The demographic, social and economic data for these 83 locales were taken from Statistics Norway (http://www.ssb.no/en/) administrative and register data at the counties (regions) level, also for the period from 2000 to 2019 and averaged over two decades, 2000‒2009 and 2010‒2019.

## Methods

To assess social vulnerability to extreme cold, this study builds on the Extremely Cold Social Vulnerability Index (ECSVI), originally developed for Arctic populations in Alaska, USA (Grigorieva et al. 2023). ECSVI is a composite measure integrating three core components: Cold Discomfort (CD) – representing physical strain caused by extreme cold exposure; Social Sensitivity (SS) – identifying population groups most at risk due to demographic, health, and socioeconomic characteristics; and Social Adaptivity (SA) – reflecting a community’s capacity to cope with and function under severe cold conditions. The SS and SA components are derived from publicly available demographic, economic, and health datasets, following established approaches to social vulnerability assessment (Cutter et al. 2003; O’Brien et al. 2004; Holand et al. 2011; Holand and Lujala 2013; Bao et al. 2015; Fekete 2019; De Pascale 2023; Luo et al. 2025).

Following standard practice, social vulnerability is determined by the interaction of exposure, sensitivity, and adaptive capacity. Building on this approach, ECSVI is formulated as:

1 $$\mathrm{ECSVI}=\mathrm{CD}+\left(\mathrm{SS}-\mathrm{SA}\right)/2$$

SS and SA are each calculated based on selected indicators (listed below), ensuring that higher SS increases vulnerability, whereas higher SA reduces it (Grigorieva et al. 2023).

### Indicator selection for social vulnerability

The first analytical step involves quantifying CD using the UTCI, an internationally recognized measure of human thermal perception integrating air temperature, humidity, wind speed, radiation, and clothing insulation, with thermal stress classified into distinct categories ranging from extreme cold to extreme heat (Jendritzky et al. 2012; Grigorieva et al. 2023). By including variables such as humidity and radiation in its calculation, the UTCI is a more comprehensive measure of cold discomfort than commonly used “wind chill” indices. For this study, exposure is defined as the number of hours per year within the two coldest UTCI categories, capturing the most hazardous thermal conditions and providing a robust basis for linking bioclimatic stress to social vulnerability patterns:

- Category 1 – Extremely cold (UTCI < − 40 °C): severe cold stress leading to rapid heat loss, frostbite, and hypothermia without adequate protection.
- Category 2 – Very cold (UTCI between − 40 °C and − 27 °C): strong physiological strain and discomfort, particularly affecting vulnerable groups such as older adults, young children, and outdoor workers.

Construction of the ECSVI requires identifying indicators that represent both sensitivity and adaptive capacity, while maintaining data accessibility and comparability across regions. Indicator selection was guided by the Arctic Social Indicator framework (Larsen et al. 2010), emphasizing data that are publicly available, temporally consistent, scalable, and relevant to Arctic social realities such as remoteness, demographic structure, and service access.

Social Sensitivity (SS) represents the degree to which population characteristics affect susceptibility to cold-related harm. Nine indicators were selected to capture demographic, socioeconomic, and infrastructural dimensions:

1. Doctor-certified sickness absence (% of employees aged 16–69)
2. Children under 4 years (% of total population)
3. Elderly (≥67 years, % of total population)
4. Unemployment rate (% of population aged 15–74)
5. Foreign-born population (% of total population)
6. Average age of municipal water pipeline systems (years)
7. Drive time to nearest emergency room (minutes)
8. Households with after-tax income < NOK 150,000 (% of all households)
9. Income inequality (Gini coefficient, EU-scale)

Adaptive Capacity, or Social Adaptivity (SA) reflects the ability of individuals and institutions to anticipate, cope with, and recover from extreme cold. Nine indicators were used to capture health, education, infrastructure, and economic resilience:

1. Municipal share of expenditures on nursing and care services (%)
2. Physicians per 10,000 inhabitants (man-years)
3. Population aged 16–66 years (% of total population)
4. Higher education attainment (% of persons aged ≥16 years)
5. Share of municipal expenditures on primary and lower secondary education (%)
6. Population connected to municipal water supply (%)
7. Green areas and sports facilities as % of residential areas (%)
8. Local roads with street lighting (%)
9. Median household income after tax (NOK)

These variables collectively represent demographic structure, economic stability, infrastructure quality, and social well-being. Higher adaptive capacity values correspond to enhanced resilience and lower vulnerability.

All indicators were normalized to a 0–1 scale using min–max normalization, allowing variables measured in different units to be directly compared while preserving their relative spatial variability. Equal weighting was applied to all indicators because no empirical or theoretical evidence justified assigning greater importance to any individual variable; this approach is widely adopted in the construction of composite social vulnerability indices (Cutter et al. 2003; Holand and Lujala 2013; Rufat et al. 2019; Grigorieva et al. 2023). Following normalization, the indicators within the Social Sensitivity (SS) and Social Adaptivity (SA) dimensions were averaged to produce composite scores, which were subsequently integrated with the Cold Discomfort (CD) component according to Eq. (1). This transparent aggregation procedure facilitates reproducibility of the ECSVI while maintaining consistency with established vulnerability assessment methodologies.

**Fig. 3:**
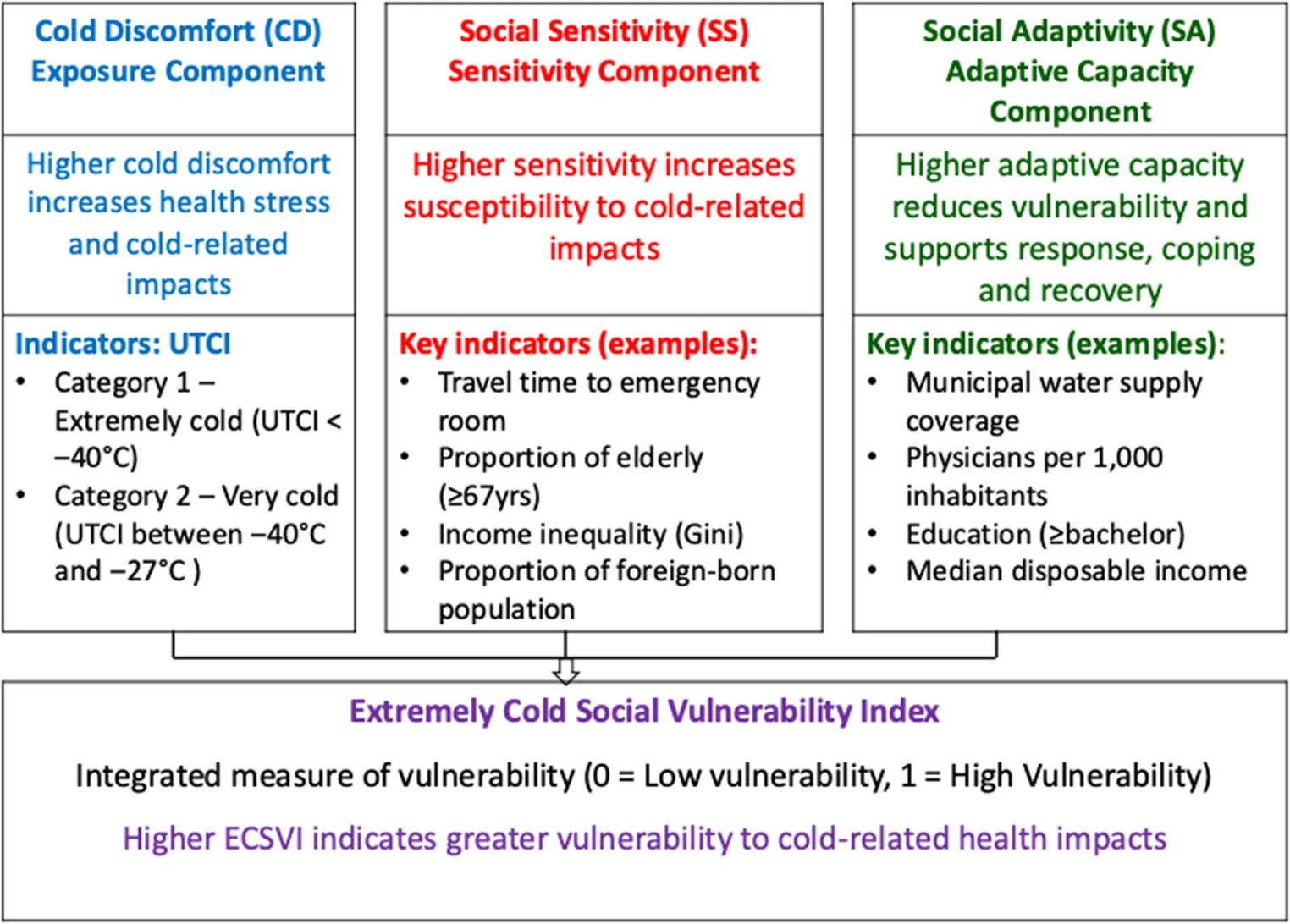
Conceptual framework of the Extremely Cold Social Vulnerability Index (ECSVI) used to assess social vulnerability to extreme cold in Northern Norway

The conceptual framework of the ECSVI is illustrated in Fig. 3. The framework highlights how climatic exposure (Cold Discomfort), demographic and socioeconomic characteristics (Social Sensitivity), and institutional and infrastructural resources (Social Adaptivity) jointly determine overall vulnerability. Rather than representing independent dimensions, these components interact to determine a community’s vulnerability to extreme cold. The framework therefore provides a transparent conceptual basis for interpreting the composite ECSVI and its spatial patterns across Northern Norway.

## Results

Spatial and temporal patterns of thermal stress across Northern Norwegian regions are represented using the annual total hours of UTCI values below − 27 °C, indicative of severe cold stress; data were aggregated for the three counties, separately for two decades: 2000–2009 and 2010–2019. Table 1 shows the highest levels of cold stress in Finnmark, and the warmest conditions in Troms.

**Table 1:** Annual number of hours with UTCI <‒27 °C in the counties of Northern Norway, 2000–2019 (hours per year)

Period/County	Norland	Troms	Finnmark
2000–2009	454	456	1011
2010–2019	482	408	883

Specifically, Finnmark experienced substantially higher annual hours of UTCI < − 27 °C than the other counties, averaging 1011 h in 2000–2009 and declining to 883 h in 2010–2019. In contrast, Nordland exhibited a slight increase in extreme cold exposure from 454 to 482 h, while Troms showed a decrease from 456 to 408 h between the two decades. Despite Finnmark’s modest temporal reductions, its greater exposure to cold stress persists.

**Fig. 4:**
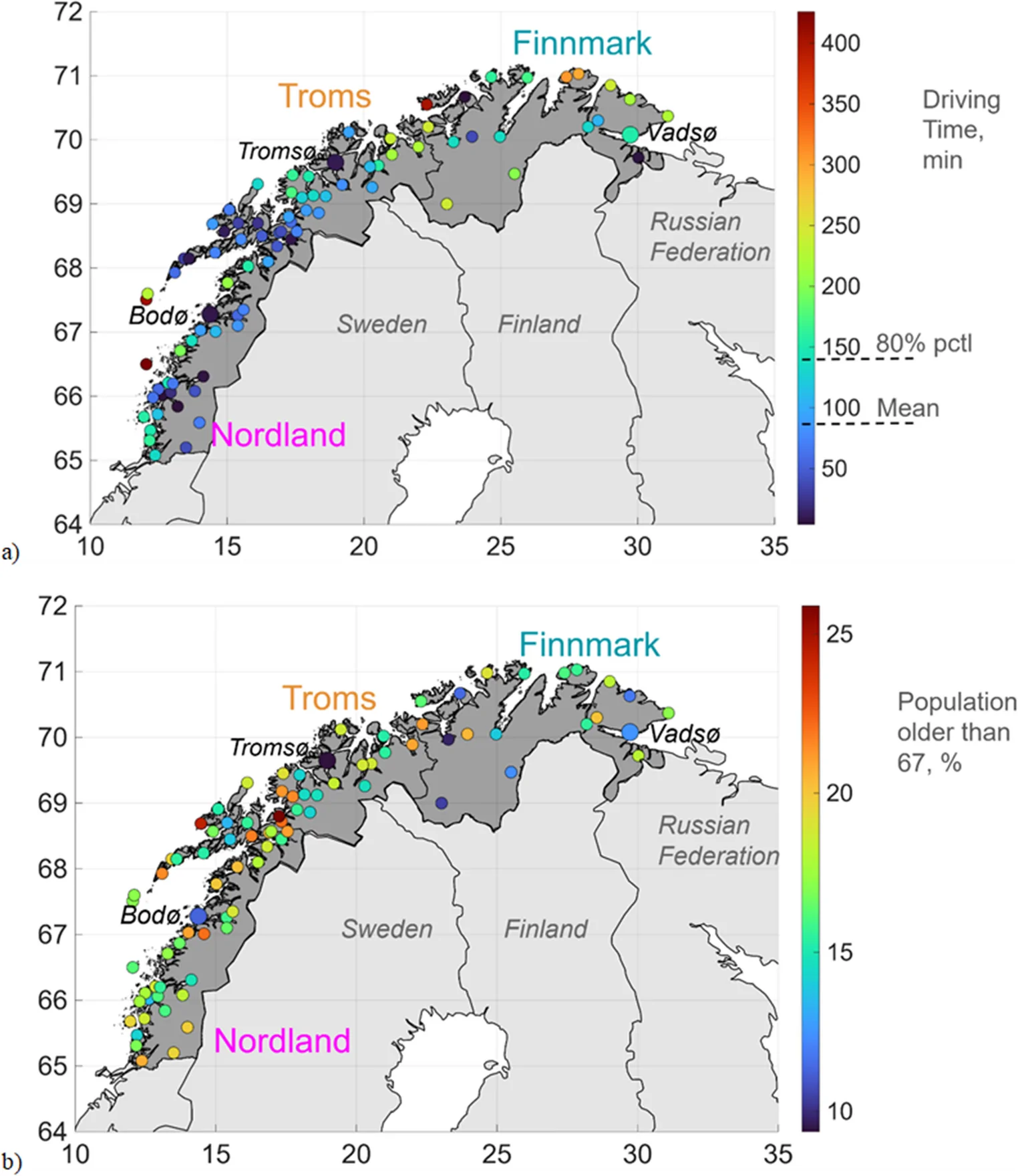
Representative social sensitivity indicators, indicating spatial drivers of vulnerability: (**a**) Drive time (min) to the nearest emergency room and (**b**) percentage of the population older than 67

**Fig. 5:**
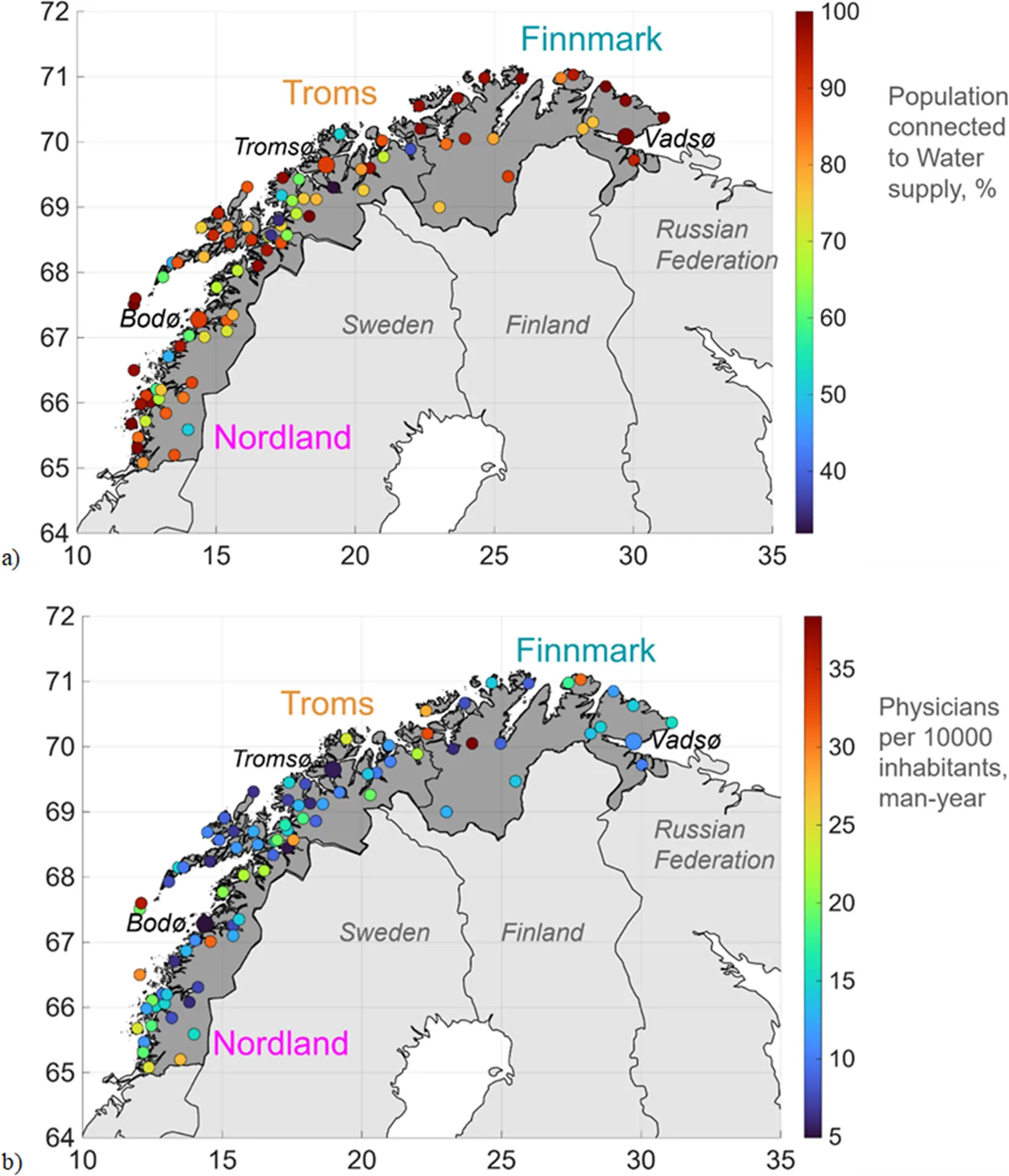
Representative adaptive capacity indicators: (**a**) % of population connected to water supply, and (**b**) Physician man-years per 10,000 inhabitants

Before discussing the fully integrated results, we first present several examples to illustrate the regional variability of the individual SS and AC metrics. Figures 4 and 5 illustrate the contributions of several indicators to the final calculation of the Social Sensitivity and Social Adaptivity.

Figure 4 shows two of the key components of SS for the period 2000–2019: (a) drive time (minutes) by road to the nearest emergency room and (b) the percentage of the total population older than 67. Long travel times to emergency healthcare coincide with remote municipalities (Fig. 4a), while higher proportions of elderly residents coincide with greater physiological susceptibility to cold-related health impacts (Fig. 4b). Together these indicators explain an important component of the elevated ECSVI observed in peripheral regions.

Travel times range from 4 to 426 min, with a mean of approximately 120 min, indicating substantial disparities in emergency response accessibility across the region (Fig. 4a). The longest drive times are concentrated in remote and sparsely populated inland and coastal areas, particularly in Nordland and Finnmark, as well as in northern Troms, reflecting infrastructural and geographic constraints. Shorter travel times are observed around major administrative centers such as Bodø, Tromsø, and Alta, where healthcare facilities are more concentrated.

Figure 4b shows the spatial distribution of the share of elderly residents over the two decades. While Fig. 4b summarizes patterns for the full period 2000–2019, our analysis distinguishes between two decades (2000–2009 and 2010–2019) to capture temporal differences as follows. The mean proportion of elderly during 2010–2019 was highest in Nordland (19%), followed by Troms and Finnmark (both 18%), compared with 17%, 16%, and 15%, respectively, during 2000–2009. Within communities, the proportion of elderly ranged from 10% to 27% in the later decade and from 9% to 25% in the earlier one, indicating a gradual and widespread demographic aging trend.

Spatial distribution of representative adaptive-capacity indicators in Fig. 5 demonstrates pronounced infrastructure and healthcare disparities between urban and remote municipalities.

Figure 5a presents the proportion of inhabitants connected to municipal water systems. As with Fig. 4, this figure displays mean values for the full period 2000–2019. However, we summarize here the results for the two decades (2000–2009 and 2010–2019) to highlight temporal change. In 2000–2009, mean connection rates were approximately 79% in Nordland, 70% in Troms, and 90% in Finnmark, rising slightly in 2010–2019 to 85%, 75%, and 92%, respectively. At the community level, the indicator ranged from 16% to 100% in the first decade and 38% to 100% in the second, showing gradual improvements but continued disparities between urban centers and remote settlements. Figure 5b illustrates spatial and temporal variations in the availability of physicians in Nordland, Troms, and Finnmark. Across communities, the number of physician man-years per 10,000 inhabitants ranged from 10 to 43, reflecting pronounced geographic disparities in healthcare provision. At the county level, the average number of physicians increased from 14, 13, and 16 in 2000–2009 to 15, 15, and 19 in 2010–2019 for Nordland, Troms, and Finnmark, respectively.

While selected indicators are presented in detail in Figs. 4 and 5, the other listed SS and SA indicators are not shown individually but are nevertheless incorporated into the composite vulnerability calculations, ensuring that all dimensions described in the methodology are reflected in the ECSVI outcomes. Taken together, the individual indicators reveal that healthcare accessibility, demographic ageing, and municipal infrastructure consistently co-occur in municipalities exhibiting the highest ECSVI values. Long travel times to emergency facilities are frequently accompanied by lower physician availability and older population structures, suggesting that these factors reinforce one another rather than act independently. Although all indicators contribute equally to the composite index by design, the spatial coincidence of these variables indicates that deficiencies in essential public services play a particularly important role in shaping regional vulnerability patterns.

**Fig. 6:**
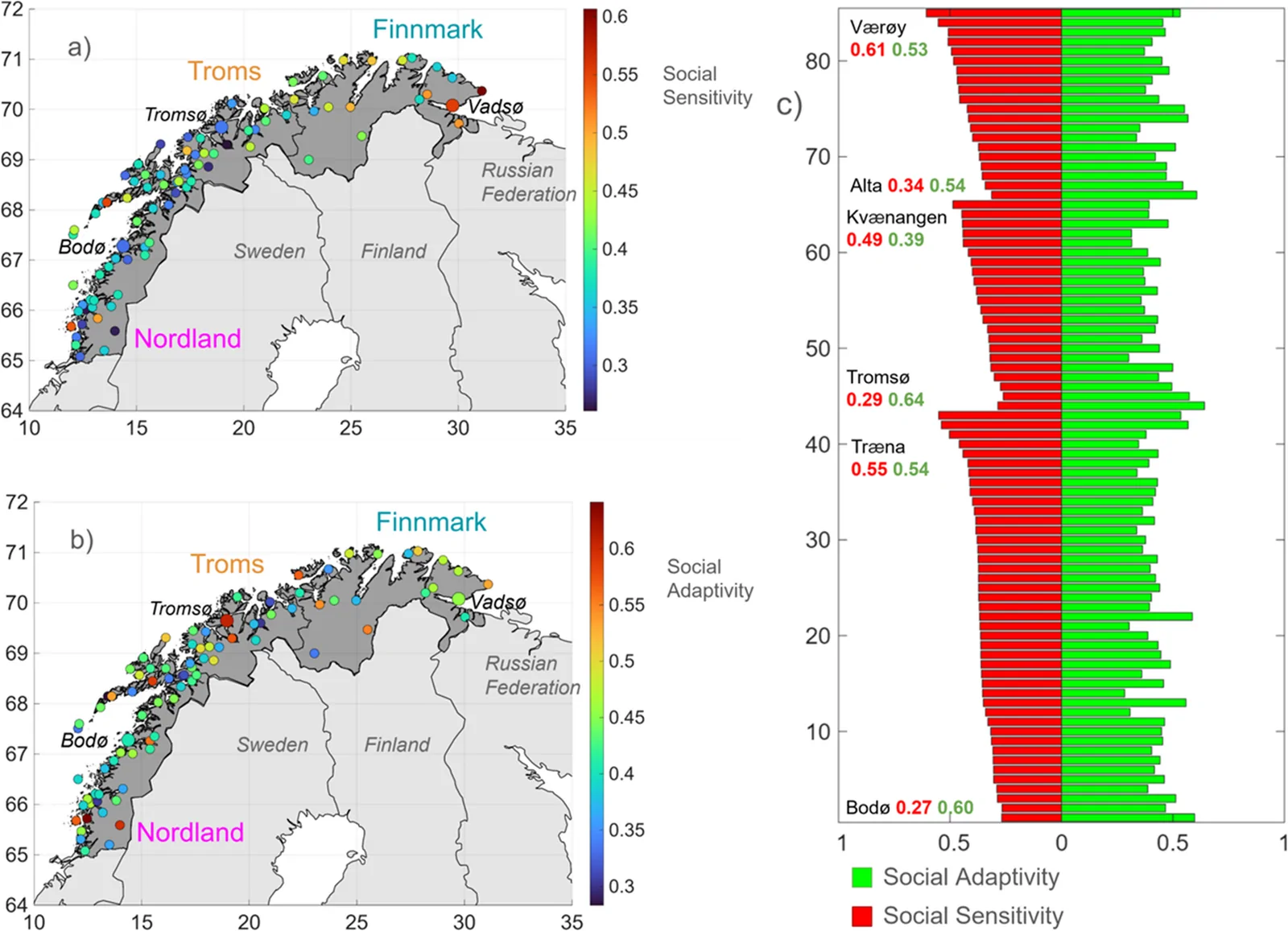
Spatial distribution of (**a**) Social Sensitivity (SS) and (**b**) Social Adaptivity, with scaling indicated by color bars. **c **shows ranked values of SS (red) and SA (green) in each region, with key communities highlighted (see text)

Figure 6a and b illustrate the spatial variations in Social Sensitivity (SS) and Social Adaptivity (SA) across the three northernmost counties, while Fig. 6c shows values ranked by SS in each county, highlighting values for selected communities representing administrative centers (Bodø, Tromsø, and Alta) and smaller settlements (Træna, Kvænangen, and Værøy). At the community level, SS values for 2000–2019 ranged from 0.27 in Bodø and 0.29 in Tromsø to 0.61 in Værøy, 0.55 in Træna and 0.49 in Kvænangen, indicating notably higher vulnerability in small and remote coastal settlements. Correspondingly, SA values ranged from 0.64 in Tromsø, 0.60 in Bodø and 0.54 in Alta to 0.39 in Kvænangen, reflecting more favorable adaptive conditions in larger administrative centers with better infrastructure, higher education levels, and greater access to public services.

**Fig. 7:**
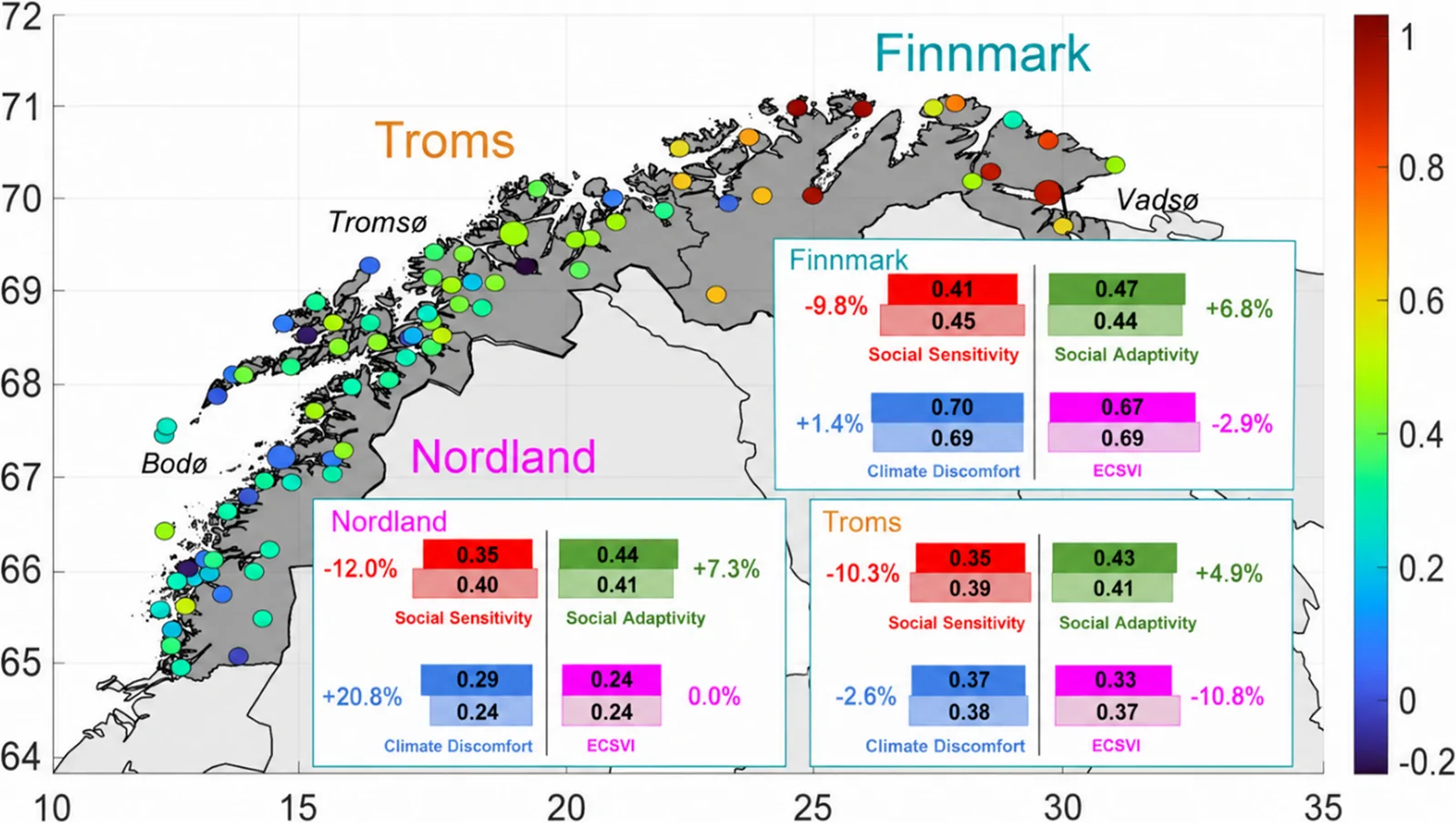
Extremely Cold Social Vulnerability Index and its components for the counties of Northern Norway. Colored circles denote averages for 2000-2019. Colored bars denote county averages of ESCVI and its components for 2000–2009 (lighter shading) and 2010-2019 (darker shading)

The temporal changes across the two decades are illustrated for the county level (Fig. 7). Social Sensitivity decreased slightly between the two decades, from 0.40, 0.39, and 0.45 in 2000–2009 to 0.35, 0.35, and 0.41 in 2010–2019 for Nordland, Troms, and Finnmark, respectively, suggesting a gradual reduction in vulnerability. Social Adaptivity increased modestly over the same period – from 0.41, 0.41, and 0.44 to 0.44, 0.43, and 0.47, linking to improvements in healthcare, education, and income levels and indicating slow but positive progress in adaptive capacity across the region. Figure 7 illustrates the aggregate effect of the CD, SS, SA in changing the ECSVI across the two decades. ECSVI values changed from 0.24, 0.37, and 0.69 in 2000–2009 to 0.24, 0.33, and 0.67 in 2010–2019 for Nordland, Troms, and Finnmark, respectively. This suggests a decline in vulnerability in Troms and Finnmark overall, although Finnmark remains the most at risk. All three components (CD, SS and SA) contributed to the decline in ECSVI for Troms, while improvements in SS and SA more than offset a slight increase of CD in Finnmark. Overall, the pattern reveals a clear spatial gradient, in which regions experiencing greater climate discomfort also tend to show higher social sensitivity and insufficient adaptive capacity, reflecting demographic ageing, economic disparities, and uneven infrastructure development over time.

The comparison between the two decades indicates gradual improvements in adaptive capacity throughout Northern Norway, although the magnitude of change differs among counties. Because the present analysis is based on decadal averages rather than annual observations, these temporal differences should be interpreted as potentially emergent trends rather than statistically robust trajectories. Future studies employing annual time series and formal trend analyses would provide a more rigorous assessment of the significance of these long-term changes.

**Fig. 8:**
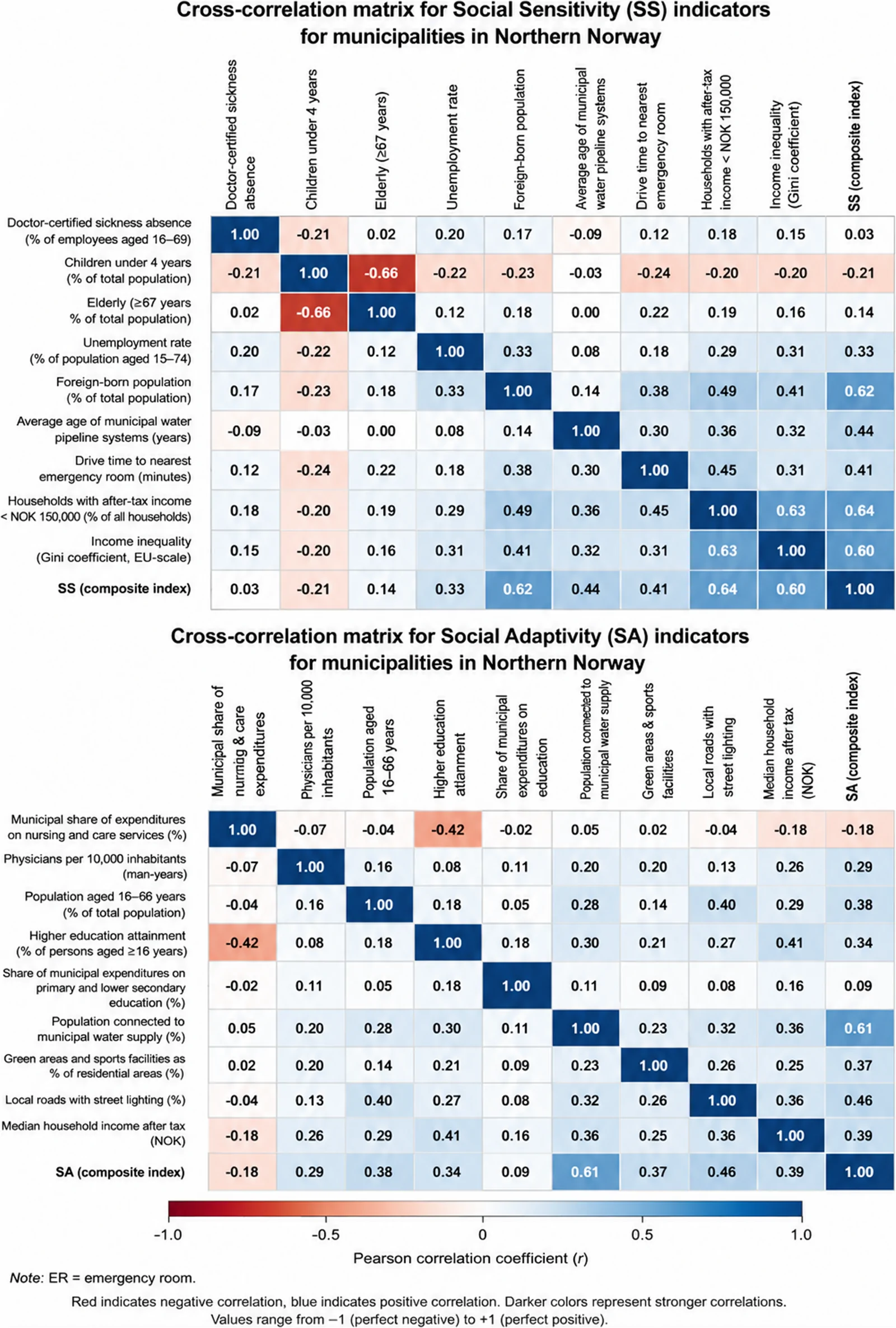
Pearson cross-correlation matrices for the Social Sensitivity (SS) and Social Adaptivity (SA) and their composite indicators across municipalities in Northern Norway

To further examine the contribution of individual indicators to the composite SS, Pearson correlation coefficients were calculated among all indicators and the resulting SS score (Fig. 8).

The strongest positive associations with SS were observed for the proportion of low-income households (*r* = 0.64), the proportion of foreign-born population (*r* = 0.62), and income inequality (Gini coefficient; *r* = 0.60), indicating that socioeconomic disparities represent the primary drivers of spatial variability in social sensitivity across Northern Norway. Infrastructure-related variables, particularly the average age of municipal water pipeline systems (*r* = 0.44), also showed a moderate contribution. Driving time to the nearest emergency room demonstrated a comparable correlation with the composite SS index (*r* = 0.41), supporting its inclusion as an indicator of accessibility-related social sensitivity and illustrating the importance of healthcare access in remote municipalities as highlighted in Fig. 4a. On the other hand, doctor-certified sickness absence exhibited only a negligible relationship with the composite index (*r* = 0.03). Pairwise correlations among indicators were generally weak to moderate, with the strongest negative association observed between the proportions of children under four years and elderly residents (*r* = − 0.66), reflecting contrasting demographic structures among municipalities. Overall, the correlation structure suggests that Social Sensitivity is primarily driven by socioeconomic conditions, while infrastructure accessibility provides an additional, complementary contribution to municipal SS.

The strongest positive association with SA was observed for the proportion of the population connected to the municipal water supply (*r* = 0.61), followed by the share of local roads equipped with street lighting (*r* = 0.46), median household income after tax (*r* = 0.39), the proportion of the working-age population (*r* = 0.38), green areas and sports facilities (*r* = 0.37), and higher educational attainment (*r* = 0.34) (Fig. 8). In contrast, municipal expenditures on primary and lower secondary education showed only a weak relationship with the composite index (*r* = 0.09), whereas the municipal share of expenditures on nursing and care services exhibited a weak negative association (*r* = − 0.18). Pairwise correlations among SA indicators were generally weak to moderate, with the strongest positive association observed between higher educational attainment and median household income (*r* = 0.41), whereas the strongest negative association occurred between municipal expenditures on nursing and care services and higher educational attainment (*r* = − 0.42). Overall, these results indicate that the selected indicators capture complementary dimensions of municipal adaptive capacity. The relatively stronger associations of infrastructure- and resource-related variables with Social Adaptivity suggest that access to municipal services and public infrastructure constitutes a key component of adaptive capacity across Northern Norway.

## Discussion

### Spatial patterns of vulnerability and adaptive capacity

The ECSVI results reveal strong spatial disparities in social vulnerability across Nordland, Troms, and Finnmark. These spatial disparities in social sensitivity reflect underlying structural characteristics of Northern Norway rather than climatic exposure alone, as remote inland municipalities exhibit higher social sensitivity due to aging populations, lower incomes, and longer travel distances to healthcare and education. Geographic remoteness, fragmented settlement patterns, mountainous terrain, and long fjord systems constrain the provision of healthcare, emergency services, and transport infrastructure, thereby reducing adaptive capacity in many peripheral municipalities. Similar spatial inequalities have been identified in previous assessments of climate adaptation in Northern Norway, where limited accessibility and demographic decline were found to increase vulnerability despite relatively high institutional capacity at the national level (Holand et al. 2011; Holand and Lujala 2013). Consequently, the ECSVI should be interpreted not only as a measure of exposure to extreme cold but also as an indicator of broader territorial inequalities in access to essential public services.

Urban centers such as Tromsø and Bodø show greater adaptive capacity, supported by diversified economies and better service provision. While vulnerability is driven more directly by exposure to cold itself, the social and infrastructural systems strongly mediate that vulnerability. This conclusion is subject to the caveat that there is a need for further testing of the sensitivity to the weights used in (1). Despite improvements in regional investments and service delivery, inequalities persist. Smaller municipalities continue to face financial constraints, limited healthcare coverage, and fragile transport systems.

Infrastructure investments implemented during the last two decades have improved healthcare provision, road connectivity, and municipal services in several parts of Northern Norway. Nevertheless, the results indicate that these improvements have not been spatially uniform. Remote municipalities continue to experience substantially longer emergency travel times and lower availability of healthcare professionals, suggesting that physical accessibility remains a critical determinant of adaptive capacity. These findings support previous studies emphasizing that effective climate adaptation in Arctic regions requires coordinated investments in transport infrastructure, healthcare accessibility, and regional development rather than relying solely on hazard-specific adaptation measures (Hovelsrud and Smit 2010; IPCC 2022).

Migration and relocation add a further adaptive dimension. In some remote areas, demographic aging, economic contraction, and climate stress drive out-migration, particularly among younger groups (Glaas et al. 2016; AMAP 2021). While migration may reduce individual exposure, it can weaken community resilience and disrupt cultural continuity, especially among Indigenous populations with deep place attachment (Larsen et al. 2010; Marino 2012; Huntington et al. 2019). Managing mobility therefore requires balancing safety and well-being with cultural preservation.

Correlation analysis (Fig. 8) further demonstrated that the selected indicators represent complementary rather than redundant dimensions of social vulnerability: the only across-indicator correlation exceeding 0.5 was between income inequality and the percentage of families with high after-tax income were correlated at higher than 0.5. Socioeconomic variables, particularly the proportion of low-income households, the foreign-born population, and income inequality, exhibited the strongest associations with the composite Social Sensitivity index, highlighting the dominant role of socioeconomic disparities in explaining spatial variability in sensitivity. In contrast, the Social Adaptivity component was primarily influenced by indicators reflecting infrastructure availability and municipal resources, especially the proportion of the population connected to the municipal water supply, the share of roads with street lighting, and median household income. These findings suggest that adaptive capacity in Northern Norway depends not only on socioeconomic conditions but also on the availability of essential public infrastructure and services.

In summary, pairwise correlations among indicators in both SS and SA remained generally weak to moderate and well below conventional multicollinearity thresholds. This indicates that the selected variables capture distinct and complementary dimensions of vulnerability rather than duplicating the same underlying information. The results therefore support the conceptual rationale for retaining equal weighting in the ECSVI while acknowledging that individual indicators contribute to the composite indices to different degrees. Future studies may further evaluate alternative weighting schemes using formal sensitivity analyses or multivariate statistical techniques, such as principal component analysis or other data-driven approaches.

### Linking sensitivity and adaptive capacity – toward transformative adaptation

The ECSVI results demonstrate that vulnerability in Northern Norway emerges from the imbalance between social sensitivity and adaptive capacity. Municipalities with higher education, income, and health infrastructure exhibit stronger resilience, while low-capacity areas remain vulnerable to cold stress (Figs. 6 and 7). Although resilience has improved overall, disparities persist between urban and peripheral communities. The implication is that effective adaptation requires integrating physical measures, such as better housing, with social investments in education, healthcare, and income stability. The ECSVI’s combined use of climatic and social indicators identifies municipalities where high exposure coincides with limited adaptive capacity, offering an evidence-based foundation for place-specific interventions and equitable distribution of adaptation resources.

Given the accelerating pace of Arctic change, transformative adaptation is necessary, moving beyond incremental improvements to structural reforms that address the root causes of vulnerability (Fazey et al. 2017). In Northern Norway, this includes embedding climate risk into regional development, diversifying economies beyond fisheries and extraction, and reinforcing health, education, and infrastructure systems (Hovelsrud and Smit 2010).

### Human dimensions and policy implications

It must be recognized that Indigenous and local knowledge is also crucial for culturally appropriate adaptation strategies. The ECSVI provides a practical tool for identifying high-risk areas and linking scientific analysis with community perspectives. Community perspectives can be incorporated through participatory vulnerability assessments, where residents contribute local knowledge on cold exposure, seasonal risks, and coping practices, improving the relevance and legitimacy of adaptation strategies (Ford et al. 2021). Co-production of knowledge with Indigenous and local communities further strengthens trust and integrates cultural understandings of climate risk into scientific analyses (Vogel and Bullock 2021). Community-based monitoring and citizen science can also complement quantitative data by documenting real-time cold impacts on infrastructure, mobility, and health (Griffith et al. 2018). Aligning quantitative data with lived experiences ensures that adaptation measures are both effective and context-sensitive, enhancing the social sustainability of Arctic communities. Embedding adaptation within community-led planning processes can enable Northern Norway to transition from reactive responses toward proactive, transformative adaptation, building Arctic societies that are resilient, inclusive, and sustainable in the face of ongoing climatic change.

From a policy perspective, the ECSVI provides a practical framework for prioritizing adaptation investments where both climatic exposure and social disadvantage are greatest. Rather than applying uniform adaptation strategies across Northern Norway, the results support geographically differentiated interventions that account for local demographic structure, healthcare accessibility, and infrastructure quality. For example, municipalities with long emergency travel times may benefit from strengthened pre-hospital emergency services, expanded telemedicine, or improved winter transport infrastructure, whereas communities characterised by demographic ageing may require targeted health monitoring and community support programmes during extreme cold events. Such place-based approaches can improve emergency response efficiency while simultaneously reducing regional inequalities in climate resilience.

### Limitations

Several limitations should be acknowledged. First, equal weighting was adopted to maximise methodological transparency and comparability with previous ECSVI applications. Although the correlation analysis (Fig. 8) indicates that the selected indicators contribute unequally to the composite indices, pairwise correlations remained generally low, supporting the inclusion of all indicators as complementary dimensions of SS and SA, alternative weighting approaches could potentially improve the representation of relative indicator importance of these social components. Future research should therefore evaluate the robustness of the ECSVI formulation in (1) under different weighting schemes, including expert-based weighting, principal component analysis, entropy weighting, or sensitivity analysis. Second, the temporal comparison relies on decadal averages and therefore identifies broad trends rather than statistically significant temporal changes. Future studies should integrate formal sensitivity analyses, uncertainty assessments, and annual time-series modelling to further strengthen the robustness and interpretability of the ECSVI framework.

## Conclusion

This study examined social vulnerability and adaptive capacity to extreme cold across counties in Northern Norway. According to the ECSVI results, Finnmark remained the most vulnerable county in both decades of the 2000–2019 study period. During this period, the ECSVI declined modestly in Troms and Finnmark and remained stable in Nordland. At the municipal level, higher vulnerability was associated with socioeconomic disadvantage, demographic ageing, and limited access to healthcare and infrastructure, whereas stronger adaptive capacity characterized larger administrative centers. These results show that climatic exposure alone does not determine cold vulnerability; its impacts are amplified or reduced by local social and infrastructural conditions.

The ECSVI provides a practical basis for geographically targeted adaptation. Priority should be given to remote and sparsely populated municipalities where high cold exposure coincides with limited adaptive capacity, particularly through improved healthcare accessibility, emergency response, transport and municipal infrastructure, and support for ageing and low-income populations. Integrating quantitative vulnerability metrics such as the ECSVI with local and Indigenous knowledge can further strengthen the relevance and equity of cold-climate adaptation in Northern Norway.

## Data Availability

Data sharing is not applicable to this article.

## References

[CR1] Adger WN (2006) Vulnerability. Glob Environ Change 16:268–281. 10.1016/j.gloenvcha.2006.02.006

[CR2] Ahmed F, Zapata O, Poelzer G (2024) Sustainability in the Arctic: a bibliometric analysis. Discover Sustain 5:121. 10.1007/s43621-024-00312-4

[CR3] AMAP (2021) Arctic climate change update 2021: key trends and impacts. summary for policy-makers; Arctic Monitoring and Assessment Programme (AMAP): Tromsø, Norwa p. 16. Available online: https://www.amap.no/documents/doc/arctic-climate-change-update-2021-key-trends-and-impacts.-summary-for-policy-makers/3508. Accessed on 14 Apr 2025

[CR4] Angell E, Flo Y, Grimsrud GM (2016) The rural and regional policy of Norway: Institutions, development features and current instruments, vol 5. Stein Rokkan Centre for Social Studies Report

[CR5] Bao J, Li X, Yu C (2015) The construction and validation of the heat vulnerability index, a review. Int J Environ Res Public Health 12:7220–7234. 10.3390/ijerph120707220 PMC4515652 26132476

[CR6] Bronfman NC, Nikole GM, Castañeda JV, Cisternas P, Repetto PB (2024) Relationship between social vulnerability and community resilience: a geospatial study in the context of natural disasters. Int J Disaster Risk Reduct 112:104774. 10.1016/j.ijdrr.2024.104774

[CR7] C3S (Copernicus Climate Change Service) (2020) Near surface meteorological variables from 1979 to 2018 derived from bias-corrected reanalysis. CDS. 10.24381/cds.20d54e34

[CR8] Cutter SL, Boruff BJ, Shirley WL (2003) Social vulnerability to environmental hazards. Soc Sci Q 84:242–261. 10.1111/1540-6237.8402002

[CR9] Czekirda J, Etzelmüller B, Westermann S, Isaksen K, Magnin F (2023) Post-little ice age rock wall permafrost evolution in Norway. Cryosphere 17:2725–2754. 10.5194/tc-17-2725-2023

[CR10] De Pascale F (2023) The social vulnerability index: a literature review. In: D’Amico S, De Pascale F (eds) Geohazards and disaster risk reduction. advances in natural and technological hazards research, vol 51. Springer, Cham. 10.1007/978-3-031-24541-1_22

[CR11] Di Napoli C, Barnard C, Prudhomme C, Cloke HL, Pappenberger F (2020) Thermal comfort indices derived from ERA5 reanalysis (version N.N). Copernicus Clim Change Service (C3S) Clim Data Store (CDS). 10.24381/cds.553b7518

[CR12] Dyrrdal AV, Isaksen K, Jacobsen JKS, Nilsen IB (2020) Present and future changes in winter climate indices relevant for access disruptions in Troms, northern Norway. Nat Hazards Earth Syst Sci 20:1847–1865. 10.5194/nhess-20-1847-2020

[CR13] Fan JF, Xiao YC, Feng YF, Niu LY, Tan X, Sun JC et al (2023) A systematic review and meta-analysis of cold exposure and cardiovascular disease outcomes. Front Cardiovasc Med 10:1084611. 10.3389/fcvm.2023.1084611 PMC10083291 37051068

[CR15] Fazey I, Moug P, Allen S, Beckmann K, Blackwood D, Bonaventura M, Burnett K, Dnson M et al (2017) Transformation in a changing climate: a research agenda. Climate Dev 10(3):197–217. 10.1080/17565529.2017.1301864

[CR16] Fekete A (2019) Social vulnerability (re-)assessment in context to natural hazards: review of the usefulness of the spatial indicator approach and investigations of validation demands. Int J Disaster Risk Sci 10:220–232. 10.1007/s13753-019-0213-1

[CR18] Ford JD, Pearce T, Canosa IV, Harper S (2021) The rapidly changing Arctic and its societal implications. Wiley Interdisciplinary Reviews: Clim Change 12(6):e735. 10.1002/wcc.735

[CR19] Glaas E, Keskitalo ECH, Hjerpe M (2016) Insurance sector management of climate change adaptation in three Nordic countries: the influence of policy and market factors. J Environ Planning Manage 60(9):1601–1621. 10.1080/09640568.2016.1245654

[CR20] Griffith DL, Alessa L, Kliskey A (2018) Community-based observing for social–ecological science: lessons from the Arctic. Front Ecol Environ 16(1):S44–S51. 10.1002/fee.1798

[CR23] Grigorieva EA (2024) Climate change and human health in the Arctic: a review. Climate 12(7):89. 10.3390/cli12070089

[CR21] Grigorieva E, Alexeev V, Walsh J (2023) Universal Thermal Climate Index in the Arctic in an era of climate change: Alaska and Chukotka as a case study. Int J Biometeorol 67(11):1703–1721. 10.1007/s00484-023-02531-2 PMC10589199 37572111

[CR22] Grigorieva EA, Walsh JE, Alexeev VA (2024) extremely cold climate and social vulnerability in Alaska: problems and prospects. Climate 12:20. 10.3390/cli12020020

[CR24] Haman F, Souza SCS, Castellani JW, Dupuis MP, Friedl KE, Sullivan-Kwantes W, Kingma BRM (2022) Human vulnerability and variability in the cold: Establishing individual risks for cold weather injuries. Temperature 9(2):158–195. 10.1080/23328940.2022.2044740 PMC9467591 36106152

[CR25] Hanna E, Francis J, Wang M, Overland JE, Cohen J, Luo D, Vihma T, Fu Q et al (2024) Influence of high-latitude blocking and the northern stratospheric polar vortex on cold-air outbreaks under Arctic amplification of global warming. Environ Research: Clim 3(4):042004. 10.1007/10.1088/2752-5295/ad93f3

[CR62] Hanssen-Bauer I, Førland EJ, Haddeland I, Hisdal H, Mayer S, Nesje A, Nilsen JEØ, Sandven S, Sandø AB, Sorteberg A, Ådlandsvik B (2017) Climate in Norway 2100 – a knowledge base for climate adaptation (NCCS Report no. 1/2017). Norwegian Centre for Climate Services (NCCS)

[CR27] Hersbach H, Bell B, Berrisford P, Hirahara S, Horanyi A, Munoz-Sabater J, Nicolas J, Peubey C et al (2020) The ERA5 global reanalysis. Q J R Meteorol Soc 146:1999–2049. 10.1002/qj.3803

[CR29] Holand I, Lujala P (2013) Replicating and adapting an index of social vulnerability to a new context: a comparison study for Norway. Prof Geogr 65:312–328. 10.1080/00330124.2012.681509

[CR28] Holand IS, Lujala P, Rød JK (2011) Social vulnerability assessment for Norway: a quantitative approach. Norsk Geografisk Tidsskrift – Nor J Geogr 65(1):1–17. 10.1080/00291951.2010.550167

[CR30] Hovelsrud G, Smit B (2010) Community adaptation and vulnerability in Arctic regions. Springer, Dordrecht. 10.1007/978-90-481-9174-1

[CR31] Hovelsrud GK, Karlsson M, Olsen J (2018) Prepared and flexible: local adaptation strategies for avalanche risk. Cogent Social Sci 4(1). 10.1080/23311886.2018.1460899

[CR32] Huntington HP, Carey M, Apok C, Forbes BC, Fox S, Holm LK, Ivanova A, Jaypoody J et al (2019) Climate change in context: putting people first in the Arctic. Reg Environ Change 19:1217–1223. 10.1007/s10113-019-01478-8

[CR33] Institute for Health Metrics and Evaluation (IHME) (2024) GBD Compare Data Visualization. Seattle, WA: IHME, University of Washington, 2024. Available from http://vizhub.healthdata.org/gbd-compare. Accessed 20 Jan 2026

[CR34] IPCC (2022) Climate change 2022: impacts, adaptation, and vulnerability. In: Pörtner H-O, Roberts DC, Tignor M, Poloczanska ES, Mintenbeck K, Alegría A, Craig M, Langsdorf S, Löschke S, Möller V, Okem A, Rama B (Eds.) Contribution of Working Group II to the Sixth Assessment Report of the Intergovernmental Panel on Climate Change. Cambridge, UK and New York, NY, USA. 10.1017/9781009325844.023

[CR35] Jendritzky G, de Dear R, Havenith G (2012) UTCI – Why another thermal index? Int J Biometeorol 56:421–428. 10.1007/s00484-011-0513-7 22187087

[CR36] Keskitalo ECH, Juhola S, Baron N, Fyhn H, Klein J (2016) Implementing local climate change adaptation and mitigation actions: the role of various policy instruments in a multi-level governance context. Climate 4:7. 10.3390/cli4010007

[CR37] Kjellman SE, Axelsson PE, Etzelmüller B, Westermann S, Sannel BK (2018) Holocene development of subarctic permafrost peatlands in Finnmark, northern Norway. Holocene 28(12):1855–1869. 10.1177/0959683618798126

[CR38] Larsen JN, Schweitzer P, Fondahl G (2010) Arctic Social Indicators (ASI)—a follow-up to the Arctic Human Development Report. In TemaNord; Nordic Council of Ministers: Copenhagen, Denmark 519. https://www.diva-portal.org/smash/get/diva2:701571/FULLTEXT01.pdf

[CR39] Lauta KC, Vendelø MT, Sørensen BR, Dahlberg R (2018) Conceptualizing cold disasters: disaster risk governance at the Arctic edge. Int J Disaster Risk Reduct 31:1276–1282. 10.1016/j.ijdrr.2017.12.011

[CR40] Lundblad MW, Broderstad AR, Njølstad I (2023) Social inequalities in health in the Arctic region: from observing to serving. Scand J Public Health 51(7):973–975. 10.1177/14034948231200515 PMC10599081 37776175

[CR41] Luo Y, Cheng X, He BJ, Dewancker BJ (2025) Identification and assessment of heat disaster risk: a comprehensive framework based on hazard, exposure, adaptation and vulnerability. Int J Environ Sci Technol 22:11275–11294. 10.1007/s13762-024-06195-2

[CR42] Malik IH, Ahmed R, Ford JD, Hamidi AR (2025) Arctic warming: cascading climate impacts and global consequences. Climate 13:85. 10.3390/cli13050085

[CR43] Marino E (2012) The long history of environmental migration: assessing vulnerability construction and obstacles to successful relocation in Shishmaref, Alaska. Glob Environ Change 22(2):374–381. 10.1016/j.gloenvcha.2011.09.016

[CR44] Melhus M, Eliassen BM, Broderstad AR (2020) From rural to urban living – migration from Sami core areas to cities in Norway. Study design and sample characteristics. Int J Circumpolar Health 79(1):1794456. 10.1080/22423982.2020.1794456 PMC7480478 32692277

[CR45] Naik N, Bot K, Whiteman G, Fleing LE, Morrissey K, Bellerby RGJ, Dupont S, Yumashev D et al (2025) A framework for assessing global health impacts of polar change: An urgent call for interdisciplinary research. Ambio. 10.1007/s13280-025-02255-0 PMC12868344 41201544

[CR46] Ni W, Stafoggia M, Zhang S, Ljungman P, Breitner S, Bont JD, Jernberg T, Atar D et al (2024) Short-term effects of lower air temperature and cold spells on myocardial infarction hospitalizations in Sweden. J Am Coll Cardiol 84(13):1149–1159. 10.1016/j.jacc.2024.07.006 39230547

[CR49] O’Brien K, Sygna L, Haugen JE (2004) Vulnerable or Resilient? A Multi-Scale Assessment of Climate Impacts and Vulnerability in Norway. Clim Change 64:193–225. 10.1023/B:CLIM.0000024668.70143.80

[CR47] OECD (2007) OECD Territorial Reviews: Norway. OECD Publishing, Paris

[CR48] OECD (2017) OECD Territorial Reviews: Northern Sparsely Populated Areas. OECD Publishing, Paris. 10.1787/9789264268234-en

[CR50] Overland JE (2024) Emergence of Arctic Extremes. Climate 12:109. 10.3390/cli12080109

[CR51] Rantanen M, Karpechko AY, Lipponen A, Nordling K, Hyvärinen O, Ruosteenoja K, Vihma T, Laaksonen A (2022) The Arctic has warmed nearly four times faster than the globe since 1979. Commun Earth Environ 3:168. 10.1038/s43247-022-00498-3

[CR52] Regional Development Trends (2025) Ministry of Local Government and Regional Development. Available at: http://www.regjeringen.no/en/dep/kdd/id504/. Accessed on October 28, 2025

[CR53] Risvoll C, Hovelsrud GK, Riseth JÅ (2022) Falling between the cracks of the governing systems: risk and uncertainty in pastoralism in Northern Norway. Weather Clim Soc 14(1):191–204. 10.1175/WCAS-D-21-0052.1

[CR54] Rocklöv J, Forsberg B, Ebi K, Bellander T (2014) Susceptibility to mortality related to temperature and heat and cold wave duration in the population of Stockholm County, Sweden. Global Health Action 7(1). 10.3402/gha.v7.22737 PMC3955771 24647126

[CR55] Rød JK, Aall C, Selseng T (2025) Towards a holistic climate service: Addressing all four climate risk determinants. Clim Serv 38:100558. 10.1016/j.cliser.2025.100558

[CR56] Rufat S, Tate E, Emrich CT, Antolini F (2019) How Valid Are Social Vulnerability Models? Annals of the American Association of Geographers. 109(4):1131–1153. 10.1080/24694452.2018.1535887 PMC11611389 39624068

[CR57] Tingstad A, Van Abel K, Bennett MM, Winston I, Brigham LW, Stephenson SR, Wilcox M, Pezard S (2025) Divergent trajectories of Arctic change: Implications for future socio-economic patterns. Ambio 54:239–255. 10.1007/s13280-024-02080-x PMC11662104 39476309

[CR63] Vennerød Ø, Schjøtt-Pedersen KE (2023) The impending population decline in northern norway: implications and demographic challenges. Arct Rev Law Politics 14:169–193. 10.23865/arctic.v14.5465

[CR59] Vogel B, Bullock RCL (2021) Institutions, indigenous peoples, and climate change adaptation in the Canadian Arctic. GeoJournal 86:2555–2572. 10.1007/s10708-020-10212-5

[CR60] Walsh JE (2021) Arctic climate change, variability, and extremes. In: Yang D, Kane DL (eds) Arctic Hydrology, Permafrost and Ecosystems. Springer, Cham. 10.1007/978-3-030-50930-9_1

